# A 600 m^2^ array of 6.5 m telescopes at the lunar pole

**DOI:** 10.1098/rsta.2023.0076

**Published:** 2024-05-09

**Authors:** Roger Angel

**Affiliations:** ^1^ Steward Observatory, University of Arizona, Tucson, AZ, USA; ^2^ Department of Optical Sciences, University of Arizona, Tucson, AZ, USA

**Keywords:** lunar pole, telescope array, exoplanet biosignatures, early universe

## Abstract

The proposed lunar telescope for optical and infrared astronomy aims at very large aperture, 600 m^2^, at a fundable cost. It comprises an array of 18 separate telescopes, each of 6.5 m aperture. The 200 m diameter array will be located within 1/2° (15 km) of a lunar pole on approximately level ground, with a perimeter screen deployed to provide shade and cooling to cryogenic temperature. The 500 m diameter screen will allow unobscured access down to 8° elevation. All 18 telescopes will reflect light into a central beam combiner to form a single image covering wavelengths from 0.4 µm to 10 µm. The initial instrument complement will include high-resolution and multi-object spectrographs to exploit the single combined field of view of two arcminute diameter, with the diffraction limited resolution of 6.5 m aperture. Scientific applications include the search for molecular biosignatures in transiting exoplanets, and the study of galaxy evolution using red-shifted spectra to beyond *z* = 10. The array cost, including delivery to the Moon by SpaceX Starship for installation using lunar base infrastructure, is around $10 billion, similar to that of the 25 m^2^ JWST. To test the concept, first a single prototype 6.5 m unit would be operated at the lunar south pole.

This article is part of a discussion meeting issue ‘Astronomy from the Moon: the next decades (part 2)’.

## 1. Background

The limitations to ground-based telescopes imposed by Earth's atmosphere are well known: spectral absorption and dispersion; image blurring (seeing) from convective turbulence; sky background from thermal emission and scattered sunlight; and thermal emission from telescope optics at around 270 K. Telescopes in space, notably the 2.4 m HST and 6.5 m cryogenic JWST, avoid many of these limitations, obtaining diffraction limited images in the optical and infrared over extended wavelength ranges. A 6.5 m, room-temperature successor in space, the Habitable Exoplanet Observatory (HabEx), is planned by NASA for launch in 2039 [1].

The Moon, with the same advantage as free space of having no atmosphere, and no greater zodiacal background than that at L2, has not been used for astronomy beyond a small, far-UV telescope placed there during the Apollo 16 mission. But given a permanent base, likely near the south pole, large telescopes could be built and operated on the Moon much like those on the Earth. An advantage over free space at the distant, L2, location of JWST is longevity. Telescopes near a permanent Moon base could be operated over many decades, benefitting in the same way as did the Hubble telescope from servicing and periodic instrument upgrades.

Current concepts for large lunar telescopes include those of Schneider, Silk and Vakili with a filled aperture of 50–100 m diameter, based on the European OWL telescope design [2]. Another concept, by Labeyrie, is for an optical interferometer hypertelescope, with many metre-class telescopes located across an impact crater of 10–25 km diameter [3].

### (a) Scientific drivers

A broad range of scientific goals has been set out for the lunar telescopes referenced above, in many instances taking advantage of the very high spatial resolution that would be enabled with 50–100 m or even 20 km aperture operated at the diffraction limit. As an example, direct imaging and spectroscopy of planets enabled by coronagraphic methods is a primary goal identified for OWL-Moon. But for other, photon-starved science goals, large telescopes that sacrifice spatial resolution in order to make affordable a very large collecting area must also be considered. Two examples of such goals are as follows.

#### i The search for biosignatures in exoplanets in the habitable zone

In the transit search method, the planet is not spatially resolved, but its atmospheric composition is revealed by absorption features that appear in the stellar spectrum during transit. It has been used with JWST to measure molecular absorption features during a transit of the exoplanet WASP 39b, a hot (1100 K), Saturn-mass planet orbiting a G8 star [4]. Spectra obtained during a 2.8-h transit revealed CO_2_ at 4.4 µm wavelength with an absorption depth of 2000 parts per million and other weaker features including H_2_O, CO and SO_2_ at 300 ppm. The sensitivity is limited to features with depths of around 100 ppm, close to the fundamental limit set by photon noise.

**Figure 1.**
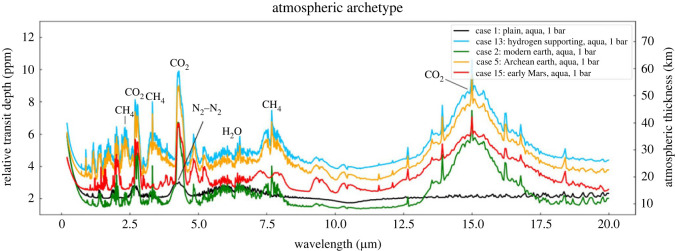
Transit spectra modelled for TOI-700 d for different atmospheric archetypes. Reproduced from [5] by permission of the AAS.

TOI-700 d is a prime transiting candidate with the same size and temperature as the Earth, transiting an M2 star at 31 pc distance. The absorption features that can be expected for different atmospheric archetypes have been modelled by Suissa *et al*. [5], and are shown in figure 1. CO_2_ at 4.4 µm is again the most prominent feature, but now with absorption depths not exceeding 10 parts per million, 10 times shallower than the limiting sensitivity for WASP 39 b. Assuming instrumental noise can be suppressed, the fundamental remaining limit set by photon noise remains, and will prohibit measurement by JWST within its lifetime. Even allowing for TOI-700 being five times brighter in the infrared than WASP 39, several hundred transits would be needed. A much larger total collecting aperture is required, and preferably also high spectral resolution to improve signal/noise ratio, by resolving individual lines in the molecular features. Then a comprehensive search for biosignatures could be undertaken of this and a range of candidates.

### (ii) Evolution of galaxies at high redshift

To characterize galaxies at high redshift, deep images with JWST have been recorded [6]. Exposures of 4 and 8 h made in three spectral bands with widths *λ*/Δ*λ* ∼ 20, covering from 3.8 to 4.8 µm revealed galaxies with redshifts in the range 5.3–9.3, at a density of approximately 10 galaxies per square arcminute. In addition, galaxies within a 2.2 arcminute deep field lensed by the cluster SMACS0723 have been studied in detail by NIRSpec spectroscopy to 5 µm, yielding redshifts ranging up to 8.498 [7].

To study spectroscopically the first galaxies at redshifts as high as *z* = 20, and supernovae to even higher redshift, broader spectral range, from the visible through to 10 µm wavelength will be needed. Multi-object spectroscopy will be valuable. But again, photon noise will be the limiting factor for the faintest objects. Spatial resolution higher than the 125 mas diffraction limit of 6.5 m aperture at 4 µm will likely not be important, given the very low surface brightness of galaxies at very high redshift.

## 2. Telescope array concept

**Figure 2.**
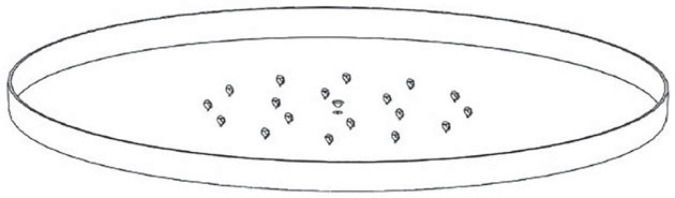
A 200 m diameter array of 18 telescopes with central beam combiner, surrounded by a sunshield.

The proposed array of 6.5 m telescopes aims at very large aperture at relatively low cost. It is sized with six telescopes at 50 m radius and 12 at 100 m to yield a total collecting area of 600 m^2^, with all the light brought to a central beam combiner. As shown in figure 2, shade and cooling to cryogenic operating temperature is provided by surrounding the array by a 500 m diameter sunshield, 23 m high and by locating the array within 1\2° (15 km) of a lunar pole, on approximately level ground.

To form a single image at the beam combiner that largely preserves etendue, for given total collecting area and lower elevation limit, the un-vignetted field size as well as the diffraction limited resolution of the combined image both vary in proportion to the diameter of the unit telescopes of the array. There is thus a high premium on configuring the array with a smaller number of larger telescopes. Hence the choice of 6.5 m apertures, a large size launchable as a monolith. Light from the individual telescopes is combined in such a way as to form a single image which maintains the diffraction limited resolution of the individual telescopes. An alternative might be to use separate instruments at each telescope and combine digital data from low noise detectors, rather than light. But at least for now, infrared detectors for high resolution spectroscopy of faint objects do not have small enough pixels and low enough read noise. Using a single rather than 18 multi-object spectrographs and high-resolution spectrographs in each waveband will greatly simplify the instrumentation and upgrading.

The preferred mirror structure is like that used for the HST primary, a monolith with an internal honeycomb sandwich. This can take advantage of the experience in manufacture of monoliths of 6.5 m and larger size developed for ground-based telescopes, including in figuring them to obtain diffraction limited images at 400 nm wavelength (15 milliarcseconds). Eighteen telescopes each of 6.5 m diameter will yield a total collecting area close to greater than 600 m^2^.

The initial instrument complement will include imagers and low- and high-resolution spectrographs covering the spectral range from 400 nm to 10 µm wavelength, including a multi-object spectrograph to exploit the 2.2 arcminute diameter field of view of the single combined image.

The goal is for the total project to cost no more than the 25 m^2^ JWST, despite its 20 times larger aperture. The enabling factors for such major cost reduction are: economies realized by mass production; the evolution of the telescope element design as experience is developed with prototype unit telescopes; the availability of a lunar base and the projected major reduction in the cost of space transportation.

The goal is to realize very large collecting area at much reduced cost by not requiring very high diffraction-limited resolution parallels of the ground-based Large Fiber Array Spectroscopic Telescope project [8]. LFAST will use a simplified array design, limited in its use to just high-resolution spectroscopy of single objects. It aims to show that 1200 m^2^ aperture can be built for a few per cent of the cost of a full-purpose, diffraction-limited, single-aperture 40 m telescope of the same area. A successful demonstration at 1200 m^2^ could lead to a fundable ground-based 20 000 m^2^ telescope, costing no more than a conventional 1200 m^2^ aperture.

LFAST will use thousands of 0.8 m telescopes, with optical fibres to bring the light of seeing limited images (1.4 arcsec) of a single star at many prime foci to the spectrograph slit. It will have only seeing-limited image resolution, but will target high-resolution spectroscopy transiting exoplanets, including the A band of molecular oxygen at 760 nm wavelength. But daylight and twilight limit its access to any given candidate to only about 20% of transits. Fibre transmission further limits its use to wavelengths less than 2 µm, while a broader spectrum of atmospheric molecules extending further into the infrared is needed to identify biosignatures unambiguously [9].

### (a) Unit telescope

**Figure 3.**
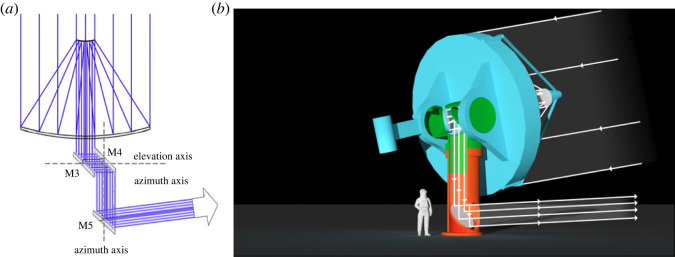
Unit telescope with 6.5 m primary. (*a*) Ray diagram. (*b*) Rendition of the telescope at the lunar pole. Parts moving with the telescope structure, including M3, are shown in blue, those moving in azimuth, including M4, in green, and stationary parts, including M5, in orange.

The proposed unit telescope, as shown in figure 3, has a monolithic 6.5 m diameter mirror, figured as a paraboloid of short focal ratio, approximately f/1. Together with a convex paraboloidal secondary in an afocal Mersenne configuration, it yields a 7-times de-magnified collimated beam, 0.93 m in diameter. The beam is remarkably free of aberrations—the field is slightly curved, but otherwise diffraction limited at 400 nm wavelength to beyond 2 arcminutes diameter.

The telescope's primary, M1, and secondary, M2, mirrors are carried by an alt-azimuth mount, along with three optical flat mirrors to direct the afocal beam to a distant beam combiner telescope. A flat tertiary mirror, M3, attached rigidly to the primary cell, directs the beam along the horizontal elevation axis. Then M4, which is rigidly attached to the elevation structure, directs the beam down the vertical azimuth axis. M5, attached to the fixed telescope base, directs the beam radially inward to a central combiner where all the beams are combined.

To minimize reflection losses and thermal emissivity, all five mirrors in the unit telescope will be silver coated. The throughput, allowing for a further three reflections at the combiner and assuming pure silver coatings, will amount to 43% at 380 nm, 66% at 500 nm and 85% at 2 µm and 10 µm.

The 18-mirror array could be adapted for interferometry by the addition of path length adjusting optics, along the lines implemented for interferometry with the four 8.2 m telescopes of the VL [10]. We do not pursue this option here, beyond noting that because of the slow rotation of the Moon, for given exposure time only relatively slow path length adjustments are required. Thus, when a full 200 m baseline is oriented perpendicular to a target of interest, maintaining zero path difference over a 1 h exposure would require a path length change of less than 2 m.

### (b) Telescope array and beam combiner

**Figure 4.**
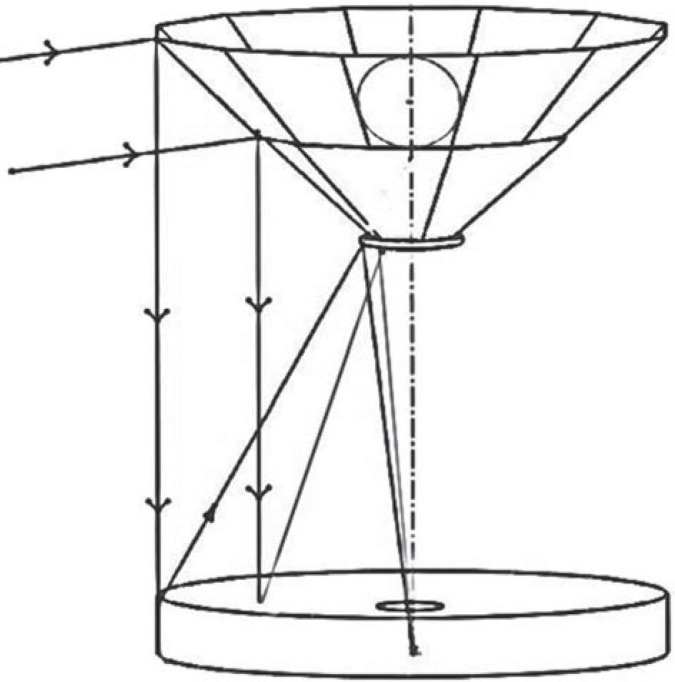
Central combiner feeding the combiner telescope, showing the path of rays from one of the 18 beams to the combined focus.

The central combiner, as shown in figure 4, is equipped with two rings of mirror facets, attached to an approximately conical support, that direct the beams from all 18 telescopes directly down into a 7 m beam combining Cassegrain telescope. The outer ring of 12 telescopes at 100 m radius feeds the upper 12 facets, and the inner ring of 6 the lower facets. Each of the facets is set parallel to the M5 mirror that feeds it, so that the downward beams are all parallel to each other and to the downward beams entering each of the M5 mirrors. Configuring the facet and M5 mirrors in this way ensures that the fields of view delivered to the beam combiner all have the same rotation angle, no matter the different azimuthal positions of the array elements. This ensures formation of a single overlapping image. The width of each facet is 1.2 m.

The diameter of the un-vignetted combined field of view realizable with this combination architecture is set by the divergence of the beams from each telescope that originate at exit pupils near the secondary mirrors. For the chosen 6.5 m aperture with 7× magnification, the exit pupil diameters are 0.93 m. The longest optical paths from the pupils of the outer ring of telescopes to the central combiner facets are 109 m. Given the 1.2 m facet size, the allowable beam spread for no vignetting, from 0.93 m to 1.2 m, is 0.27 m. This corresponds to 510 arcsec for the relay beams, and thus 2.1 arcminutes on the sky (allowing for 7× magnification). This realizes our target of obtaining an unvignetted combined field similar to the deep, 2.2 arcminutes field of JWST.

## 3. Location with sunshade at the lunar pole

The array should be located within 1/2° in latitude (15 km) from the lunar pole, so as to minimize the sunshade height and thus maximize the sky available for observation. Figure 2 illustrates the 200 m diameter array surrounded by a cylindrical thermal shield 500 m in diameter and 23 m high, This height is chosen so that, at a location 1/2° from the pole, the sun, at its highest elevation of 2°, does not illuminate the top of the most distant telescope of the array, 350 m away, even when the telescope is zenith pointed. It is also low enough that the nearest telescopes, when viewing at 8° elevation, have an unobscured view over the shield. The shield may be lowered when necessary to warm the telescope during construction, servicing or upgrades.

During local ‘summer', when the sun is at its highest elevation, the inside of the shade across from the sun will be illuminated. The inner surface will be silvered and the shade tilted back by 3° to reflect the sunlight, away from the telescopes and out of the cylinder interior.

Seen from the pole, the Earth is about 100 times brighter than the full moon seen from Earth, and rises as high as 8° above the horizon, always from about the same direction. To minimize scattered light, the primary and secondary mirrors will be shielded by baffles and non-time critical observations made at the time in the month when the target orientation minimizes scattered Earthlight.

## 4. Construction and transportation

### (a) Primary mirrors and alt-az mounts

Made in the same way as most large monolithic primary mirrors used in ground-based telescopes, the 6.5 m primary mirrors of the array will be cast from borosilicate glass with honeycomb sandwich structure. Some 20 telescope primary mirrors of this type, 6.5 m or 8.4 m diameter, have been made at the University of Arizona Richard Caris Mirror Laboratory, for the MMT, the LBT, the Rubin telescope, the Magellan and Giant Magellan telescopes, and others.

The 6.5 m, f/1 mirrors for the array will be figured with rms surface error ≤14 nm, in order to obtain diffraction limited images down to 400 nm wavelength. Such high accuracy in highly aspheric surfaces is within the capability of currently proven methods of metrology and figuring. For example, the first of the six 8.4 m diameter, off-axis segments of the f/0.7, 25 m primary mirror being made at the Mirror Lab for the Giant Magellan telescope, was finished to 14 nm rms surface error.

In ground telescopes, thermal stability of borosilicate glass mirror figure is ensured by ventilation with air at ambient temperature, close to the temperature during fabrication. In space, the mirrors must have accurate figure after cryogenic cooling. Fortunately, the thermal expansion coefficient of the E6 borosilicate glass used for the existing mirrors is much reduced on cooling to cryogenic temperature, from 2.8 × 10^−6^/°C down to 0.5 × 10^−6^/°C at 50 K and 0.2 × 10^−6^/°C at 40 K, similar in magnitude to the coefficients of Zerodur and ULE glass at the same temperatures. The overall contraction on cooling results in shrinkage of 0.06%, but because the composition and expansion coefficient of E6 glass is controlled to very high accuracy (1 part in 10^8^), large figure errors from contraction are not expected. The mirrors in their cells will be tested at their cryogenic operating temperature, and refigured if needed to ensure high figure accuracy in operation. The secondary may be made with active shape control if needed, to correct for time-variable aberrations.

The mirror support cell will be optimized to accommodate differential expansion between the mirror and cell. The cell structure may be engineered to contract by the same amount as the glass on cooling from room temperature, for example, by using Invar, which has about the same overall fractional contraction of 5 × 10^−4^.

The mass of the 6.5 m honeycomb mirrors made for ground-based telescopes is 10 tons (310 kg m^−2^). For use in space, lighter weight construction approaches have been designed and analysed [11]. For a 4 m monolith, an areal density of 94 kg m^−2^ may be achieved by using rib, faceplate and backplate thicknesses all half that of current production, hexagonal cell spacing slightly more than twice as large, and mirror thickness that tapers down toward the outer edge. A 1 m test casting has been made with a specific mass of 180 kg m^−2^ (the same as the HST honeycomb mirror) [11]. Meniscus monoliths are also a possibility; those used in ground-based telescopes have areal densities of 210 kg m^−2^ (4.3 m Lowell Discovery Telescope) and 417 kg m^−2^ (8.2 m VLT). Our baseline design for the 6.5 m mirrors is a honeycomb sandwich weighing 155 kg m^−2^, for a primary mirror mass of 5 tons.

The total mass of all elements of the unit telescope is an important consideration for transportation to the Moon. The mass of the 6.5 m cell will be reduced from the 20 tons of steel used on the ground to 10 tons, and the mass of the alt-az mount and drive elements will be limited also to 10 tons, for a total unit telescope mass, including the 5- ton mirror, of 25 tons.

### (b) Transportation

It is envisaged that launch and delivery of 19 telescopes (including the combiner telescope) to the Moon's surface will be by SpaceX Starship [12], which has both the launch mass capacity (100 tons) and the payload size (8 m diameter×16 m long) to carry four telescopes at a time. The telescopes will be packaged in the form of large sub-assemblies. The mirrors in their support cells will be the heaviest and largest of these assemblies, 15 tons each, 8 m in diameter and 1.5 m thick. The cell assemblies will be configured and stacked to survive launch loads as well as operation at cryogenic temperature in the 1/6 g environment of the Moon. We will suppose that the telescope foundations will be made largely from local materials, but with some 10 tons per telescope of additional materials from Earth. The total mass to be transported will then be some 800 tons.

## 5. Discussion

The viability of this lunar array concept depends on two critical factors: the capability to transport the telescopes to the Moon at affordable cost, and the availability of a 500 m site within 15 km of a pole, with a base nearby having the infrastructure and capability to build the array. The landing site and associated base should be over the horizon from the telescope array, approximately 50 km distant, to avoid damage from ejected regolith.

Regarding cost, we will suppose that a realistic limit to funding for a project of this type would be 10 billion, similar to the JWST, and that up to a third of this total could be made available for transportation of the 800 ton total mass to the lunar pole. This corresponds to about 400 million available for each of the eight 100 ton payloads delivered to the lunar surface. SpaceX indicates that Starship will be able to deliver 100 ton payloads to the Moon, by refuelling in orbit which would require an additional eight fuel launches. If nine launches are needed to deliver 100 tons to the Moon, at the cost projected by SpaceX of 90 million. Thus, we could survive a factor of four increase in individual Starship launch cost, to $40 million, but not much more.

This paper has focused specifically on a means to obtain a very large collecting area on the Moon, sacrificing the potential for larger, coherent aperture to increase spatial resolution. But if indeed it becomes affordable to deliver finished 6.5 m mirrors to the Moon at the costs estimated above, then higher resolution configurations must also be considered, beyond adding delay lines to the 18-telescope array, as noted above. For example, four cold 7 m mirrors could be configured on a single mount to give five times the diffraction-limited resolution of JWST, in addition to deep star suppression by double nulling [13]. It is beyond the scope of this paper to go into detail, but we note that such a telescope would have the sensitivity to search the full HabEx target list [14] for biosignatures, in the infrared as well as the optical.

Given the remarkable and specific improvements in observational power that could be made using telescopes at the Moon's pole, it will be important for astronomers to consider and develop their full scientific potential. This could then add motivation to establishing a polar lunar base with the infrastructure necessary to build the array, and to protecting the site for astronomy within 15 km of the pole. The base would undertake tasks including surface preparation and installing foundations, as well as telescope construction that includes handling the 15 ton mass mirror assemblies. Longevity of the base over many decades will be very valuable, enabling maintenance, instrument upgrades and replacement of expendables.

Much could be learned early on about prospective polar sites as part of the Artemis project, with studies of cooling by a deployable solar shield, the level and rate of dust contamination at the pole and the depth of bedrock below the regolith layer. Later, a 6.5 m prototype telescope would be built and tested, first here on the Earth, then at the site with a deployable sunshade. This prototype will be critical to demonstrate that a 25 ton telescope can be made and installed at the pole, and to assess optical and mechanical performance while operating at cryogenic temperature.

## Data Availability

This article has no additional data.
